# Case Report: Efficacy of transcutaneous spinal cord stimulation combined with overground robotic-assisted gait training in chronic incomplete spinal cord injury

**DOI:** 10.3389/fnhum.2026.1795068

**Published:** 2026-05-18

**Authors:** Gobinathan Chandran, Nur Shafawati Kamsani, Ning Tang, Hwa Sen Lai, Tracy Ong, Su-Ann Cheng, Jeong Hoon Lim, Ravi Shankar, Effie Chew

**Affiliations:** 1 Division of Rehabilitation Medicine, Department of Medicine, National University Hospital, Singapore, Singapore; 2 Department of Rehabilitation Medicine, Alexandra Hospital, Singapore, Singapore; 3 Department of Rehabilitation, Alexandra Hospital, Singapore, Singapore; 4 Department of Rehabilitation Medicine, Tan Tock Seng Hospital, Singapore, Singapore; 5 Department of Medicine, Yong Loo Lin School of Medicine, National University of Singapore, Singapore, Singapore; 6 Medical Affairs – Research Innovation & Enterprise, Alexandra Hospital, Singapore, Singapore

**Keywords:** case report, gait rehabilitation, neuromodulation, robotic-assisted gait training, spinal cord injury, transcutaneous spinal cord stimulation

## Abstract

**Background:**

Spinal cord injury (SCI) causes significant motor, sensory, and autonomic dysfunction, with gait recovery being a primary rehabilitation goal. Transcutaneous spinal cord stimulation (tSCS) combined with robotic-assisted gait training (RAGT) represents a promising synergistic approach to enhance motor recovery, though evidence for overground exoskeletal training with tSCS remains limited.

**Case presentation:**

We report a 40-year-old male with chronic incomplete traumatic SCI (T5, ASIA D) who underwent a two-phase intervention: Phase I consisted of 16 sessions of overground RAGT using the EksoGT exoskeleton, followed by Phase II comprising 16 sessions of RAGT combined with tSCS using the ARC-EX system. Comprehensive assessments included ISNCSCI neurological examination, 10-meter walk test (10MWT), spatiotemporal gait analysis, surface electromyography (EMG), and central motor conduction time (CMCT).

**Results:**

The participant demonstrated substantial improvements in gait speed from 0.26 m/s at baseline to 0.41 m/s at four-week follow-up (Δ = +0.15 m/s), exceeding the minimal clinically important difference of 0.13 m/s. Lower Extremity Motor Score improved from 35/50 to 42/50, with notable distal lower limb gains. Cadence increased from 31.53 to 39.74 steps/min (right) and 33.29 to 38.67 steps/min (left), while stride length improved from 0.91 m to 1.17 m (right) and 0.95 m to 1.17 m (left). EksoGT MinAssist values decreased from an average of 61 (Phase I) to 50 (Phase II), indicating greater active participant engagement with tSCS. EMG recordings demonstrated enhanced neuromuscular activation following tSCS, with more organized muscle recruitment patterns. CMCT decreased from 21.9 ms to 19.7 ms on the right side, suggesting improved corticospinal tract conduction. EQ-VAS improved from 80 to 90. No adverse events were reported.

**Conclusion:**

This is the first reported case integrating tSCS with overground exoskeletal training in SCI rehabilitation. The combination of tSCS and RAGT appears safe and may provide synergistic benefits for motor recovery, particularly when conventional therapy reaches a plateau. These findings support further investigation through larger controlled studies to validate optimal stimulation protocols and generalizability.

## 1 Introduction

Spinal cord injury (SCI) is a devastating neurological condition that disrupts motor, sensory, and autonomic functions, affecting approximately 15.4 million people worldwide as of 2021 (Margetis et al., 2025; WHO, 2025). Despite preserved neurological function below the lesion level, 30–40% of patients with incomplete SCI remain non-ambulatory or dependent on assistive devices in the chronic phase (Jeawon et al., 2023; Kirshblum et al., 2021; Lam et al., 2007), making gait recovery a primary rehabilitation priority (Anderson, 2004). Furthermore, physical inactivity following SCI contributes to adverse cardiovascular and metabolic health outcomes (Jacobs and Nash, 2004; Warburton et al., 2007). Addressing these challenges requires innovative rehabilitation strategies that can enhance motor recovery beyond conventional approaches.

Robotic-assisted gait training (RAGT) is an established rehabilitation strategy that enables intensive, repetitive, task-specific locomotor practice for individuals with SCI. Among available systems, overground exoskeletal devices such as the EksoGT offer distinct advantages over treadmill-based platforms, including exposure to variable environmental challenges, natural ground reaction forces during full weight-bearing, and greater ecological validity for transfer to community ambulation (Bach Baunsgaard et al., 2018; Gil-Agudo et al., 2023; Park et al., 2024). RAGT has been shown to promote neuroplasticity and cardiometabolic health through repetitive weight-bearing locomotion (Kozlowski et al., 2015). However, functional recovery in chronic SCI often plateaus with conventional training approaches alone, highlighting the need for adjunctive strategies to augment rehabilitation outcomes.

Transcutaneous spinal cord stimulation (tSCS) is a more recent, primarily research-based neuromodulation technique that has shown growing promise as such an adjunct. By delivering electrical stimulation non-invasively through surface electrodes placed over the spinal cord, tSCS can tonically increase the excitability of spinal locomotor circuits, thereby lowering the threshold for voluntary activation of residual motor pathways (Gerasimenko et al., 2015; Hofstoetter et al., 2013). Multiple studies have demonstrated that tSCS can augment voluntary locomotor activity in individuals with motor-incomplete SCI (Hofstoetter et al., 2015), reduce spasticity through modulation of pre- and postsynaptic inhibition (Hofstoetter et al., 2014; Minassian et al., 2024; Alashram et al., 2023), and enhance motor control, trunk stability, and quality of life (Megía García et al., 2020; Samejima et al., 2022). Importantly, no serious adverse events attributable to tSCS have been reported across these studies, supporting its safety profile for clinical application (Shankar et al., 2025b).

While both tSCS and RAGT have independently shown promise in SCI rehabilitation, their combined application—particularly with overground exoskeletal systems—remains largely unexplored. While a recent RCT by Comino-Suárez et al. (2025) demonstrated the efficacy of tSCS combined with treadmill-based RAGT, no study has evaluated this combination with overground exoskeletal systems. The present single-case study provides the first detailed clinical description of this novel pairing. While the RCT by Comino-Suárez et al. (2025) established efficacy for tSCS with treadmill-based RAGT, the present case offers unique contributions including comprehensive neurophysiological (EMG, CMCT), biomechanical (spatiotemporal gait analysis), and patient-reported outcome data for the overground exoskeletal paradigm, which complement group-level findings and inform the design of future overground-specific trials.

We hypothesize that the addition of tSCS to overground RAGT (Phase II) will result in greater improvements in gait speed (as measured by the 10MWT), spatiotemporal gait parameters, and lower extremity motor scores compared to RAGT alone (Phase I) in this participant with chronic incomplete SCI. This case report presents the first documented experience of combining tSCS with overground exoskeletal training in a patient with chronic incomplete SCI. Given the single-case design, this study adopts a proof-of-concept approach to evaluate the safety, feasibility, and preliminary efficacy of this combined intervention.

## 2 Case description

### 2.1 Patient information

The participant was a 40-year-old male with no significant medical comorbidities who sustained a traumatic spinal cord injury in a road traffic accident approximately 3 years prior to study enrollment (chronic phase). The study intervention was delivered in an outpatient rehabilitation setting. Initial imaging revealed burst fractures at the T3–T4 vertebrae with severe spinal cord compression. Emergency surgical management included T3–T4 decompressive laminectomy and posterior spinal instrumentation spanning T2–T6. Postoperatively, the injury was classified as incomplete SCI with a neurological level of T5 and an American Spinal Injury Association Impairment Scale (ASIA) grade D.

Following acute rehabilitation spanning approximately 5 months, the participant achieved modified independence in basic activities of daily living and community ambulation with bilateral elbow crutches.

At study enrollment, the participant presented with residual lower limb weakness, sensory impairment, and spasticity affecting bilateral hamstrings and gastrocnemius. Baseline functional characteristics are presented in Table 1. At the time of study enrollment, the participant was not receiving any structured physiotherapy or formal rehabilitation program. His usual care comprised self-directed home exercises and community ambulation with bilateral elbow crutches. No concurrent rehabilitation interventions were received during the study period apart from the study protocol.

**Table 1:** Timeline showing the episode of care with relevant data points.

Outcome measure		Baseline		Post-Phase I		Post-Phase II		4 weeks after Phase II	
		Right	Left	Right	Left	Right	Left	Right	Left
ISNCSCI-NLI		T5		T5		T6		–	
ISNCSCI-AISA		D		D		D		–	
ISNCSCI-Sensory	Light touch	49	49	51	51	51	51	–	
	Total light touch	98/112		102/112		102/112		–	
	Pin prick	43	43	45	46	47	48	–	
	Total pin prick	86/112		91/112		95/112		–	
ISNCSCI-Motor	Hip flexors (L2)	3	4	3	4	3	4	–	
	Knee extensors (L3)	4	5	5	5	5	5	–	
	Ankle dorsiflexors (L4)	3	2	5	3	5	3	–	
	Long toe extensors (L5)	4	0	5	1	5	2	–	
	Ankle plantar flexors (S1)	5	5	5	5	5	5	–	
	Total LEMS	35/50		41/50		42/50		–	
MTS (R2-R1)	Quadriceps	0	12	0	0	0	0	0	0
	Hamstrings	53	20	50	40	50	15	0	0
	Gastrocnemius	32	25	40	30	35	30	17	13
Functional mobility	Gait speed from 10MWT (m/s)	0.26		0.29		0.37		0.41	
	TCT (out of 24)	24		24		24		24	
	WISCI-II (out of 20)	16		16		16		16	
Gait analysis by MobileGait	Cadence (steps/min)	31.53	33.29	30.23	29.98	36.51	36.70	39.74	38.67
	Stride length (m)	0.91	0.95	1.00	0.99	1.06	1.07	1.17	1.17
	Step length (m)	0.41	0.50	0.54	0.46	0.54	0.52	0.62	0.56
	Stride time (s)	3.81	3.61	3.97	4.00	3.29	3.27	3.02	3.10
	Step time (s)	1.77	2.04	1.88	2.12	1.60	1.69	1.45	1.57
CMCT (ms)		22.9	20.9	21.9	20.1	19.7	20.3	–	–

ISNCSCI, International Standards for Neurological Classification of Spinal Cord Injury; NLI, neurological level of injury; ASIA, American Spinal Injury Association; LEMS, Lower Extremity Motor Score; MTS, Modified Tardieu Scale; ROM, range of motion; CMCT, central motor conduction time; 10MWT, 10-meter walk test; TCT, Trunk Control Test; WISCI-II, Walking Index for Spinal Cord Injury II.

### 2.2 Timeline

**Figure 1:**
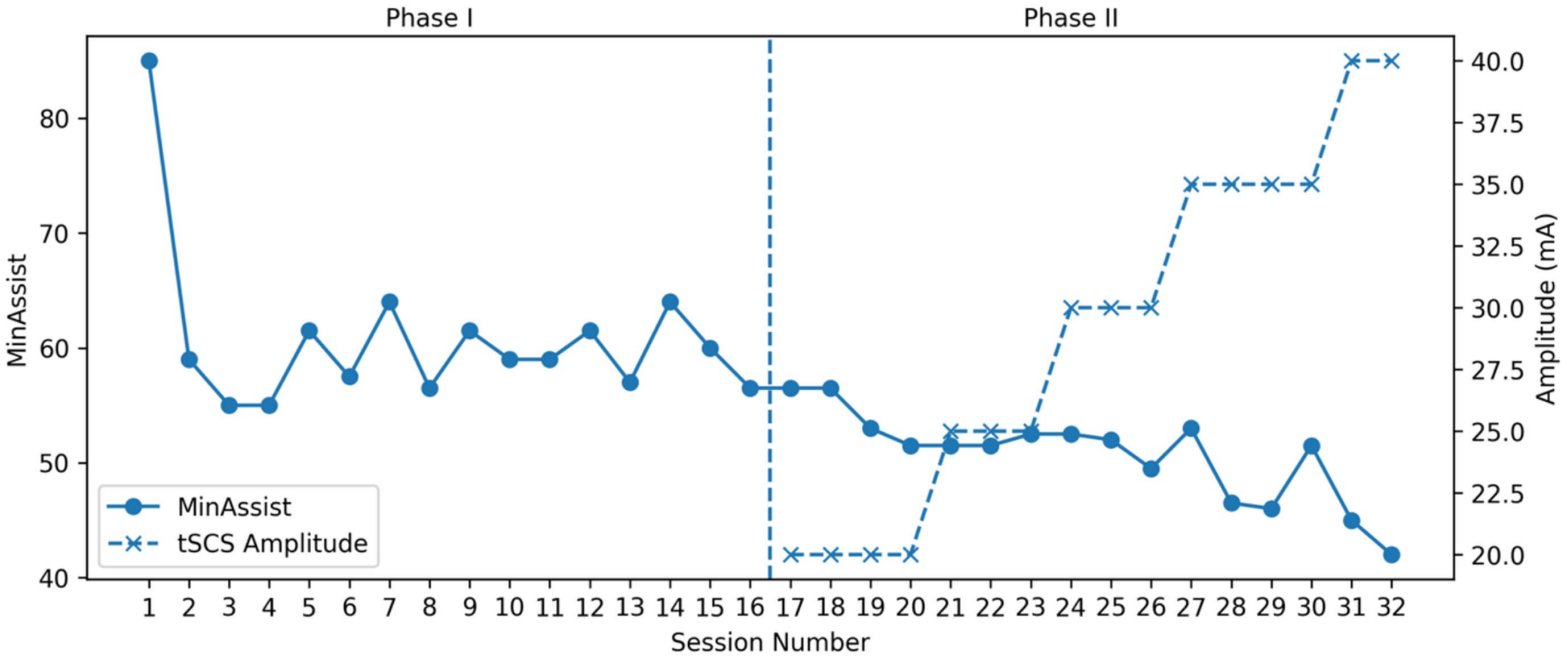
Sequential representation of training sessions across Phase I and Phase II: Sessions are displayed continuously from Session 1 to Session 32 to reflect the true sequential study design. Phase I (Sessions 1–16) consisted of robotic-assisted gait training alone, while Phase II (Sessions 17–32) included concurrent transcutaneous spinal cord stimulation (tSCS). The dashed vertical line indicates the transition between Phase I and Phase II. MinAssist values demonstrate progressive reduction in robotic assistance, while tSCS amplitude (mA) is shown for Phase II sessions only.

The study intervention was structured in two sequential phases over a 17-week period. Phase I (Weeks 1–8) consisted of 16 sessions of overground RAGT using the EksoGT exoskeleton combined with conventional physiotherapy, delivered at a frequency of two sessions per week. The participant attended all 16 scheduled sessions in each phase (100% adherence). Following a one-week break (Week 9) for Post-phase 1 assessment, during which the participant rested at home without any structured physiotherapy, Phase II commenced. The training progression, including MinAssist values guiding assistance reduction, is illustrated in Figure 1. Phase II (Weeks 10–17) comprised 16 additional sessions of the same RAGT protocol supplemented with concurrent tSCS. Outcome assessments were conducted at four time points: baseline, post-Phase I, post-Phase II, and at 4 weeks post-intervention follow-up (Table 1).

## 3 Diagnostic assessment

Comprehensive baseline evaluation confirmed the diagnosis of chronic incomplete traumatic SCI. Neurological examination using the International Standards for Neurological Classification of Spinal Cord Injury (ISNCSCI) revealed a neurological level of injury (NLI) at T5 with ASIA Impairment Scale grade D (Kirshblum et al., 2011). Motor assessment demonstrated Lower Extremity Motor Score (LEMS) of 35/50, with notable weakness in hip flexors (L2: 3/5 right, 4/5 left), knee extenors (L3: 4/5 right, 5/5 left), ankle dorsiflexors (L4: 3/5 right, 2/5 left), and long toe extensors (L5: 4/5 right, 0/5 left). Sensory examination showed light touch score of 98/112 and pinprick score of 86/112.

Spasticity assessment using the Modified Tardieu Scale (MTS) revealed increased tone in bilateral lower extremities, At baseline, MTS (R2–R1) revealed no spasticity in the right quadriceps and mild spasticity on the left (12°). Greater spasticity was observed in the hamstrings (right: 53°, left: 20°) and gastrocnemius (right: 32°, left: 25°), with a right-sided predominance. Functional mobility evaluation demonstrated preserved trunk control (Trunk Control Test: 24/24) and walking independence with assistive device (Walking Index for Spinal Cord Injury II [WISCI-II]: 16/20). Gait analysis using the MobileGait system (Shi et al., 2020, 2022) documented baseline spatiotemporal parameters including reduced cadence (31.53 steps/min right, 33.29 steps/min left), prolonged stride times, and asymmetric hip range of motion, measured using the MobileGait system’s kinematic analysis. Compared to age-matched normative values reported in a South-East Asian population (gait speed ~1.10 to 1.17 m/s; cadence ~109 to 119 steps/min) (Lau et al., 2020), the participant demonstrated markedly reduced cadence (~31 to 39 steps/min) and walking speed (0.26–0.41 m/s), corresponding to reductions of approximately 60–70% and 65–75% below normative values, respectively. These findings are consistent with significantly impaired locomotor function following incomplete spinal cord injury. Baseline hip range of motion was 41.1° on the right and 53.9° on the left during the gait cycle. These values were above normative ranges reported in healthy adults (~30 to 40° of sagittal plane excursion during gait) (Neumann, 2017; Perry and Burnfield, 2010). The increased excursion, particularly on the left, may reflect compensatory strategies associated with impaired gait mechanics and could contribute to the observed prolonged stride times.

## 4 Therapeutic intervention

All sessions were supervised by a physiotherapist with 7 years of experience in SCI rehabilitation and robotic-assisted gait training. Each conventional physiotherapy session lasted 60 min.

### 4.1 Phase I: robotic-assisted gait training

The participant underwent 16 sessions of overground RAGT using the EksoGT exoskeleton (Ekso Bionics, USA) combined with conventional physiotherapy over 8 weeks. The EksoGT is a powered lower-limb exoskeleton that provides variable motor assistance to facilitate overground walking. The EksoGT overground exoskeleton was selected over treadmill-based systems to provide ecologically valid gait training with natural weight-bearing, variable speed, and environmental interaction, which may better facilitate transfer to community ambulation (Gil-Agudo et al., 2023). Conventional physiotherapy included strengthening exercises targeting bilateral lower extremities, balance training and overground gait training without the exoskeleton. Each session lasted for 1 h. Training sessions focused on gait practice with progressive reduction of robotic assistance as tolerated, guided by the EksoGT MinAssist scores, where lower values indicate reduced motor assistance and greater active patient engagement.

### 4.2 Phase II: combined RAGT and transcutaneous spinal cord stimulation

Following a one-week mid-trial break, Phase II consisted of 16 sessions of the same RAGT protocol supplemented with concurrent tSCS using the ARC-EX™ system (ONWARD Medical, Switzerland). The one-week break was chosen to allow mid-point assessment of any learning or training effects from Phase I while minimising deconditioning. Stimulation parameters included a frequency of 30 Hz with 1 ms pulse width using a charge-balanced biphasic waveform. Stimulation was applied continuously throughout the RAGT component of each session for a total duration of 45 min per session. Surface electrodes were positioned with cathodes at the T10 and L1 vertebral levels and anodes placed bilaterally over the iliac crests. Stimulation amplitude was initiated at 20 mA and progressively titrated upward across sessions based on participant comfort and tolerance, motor response (assessed via MinAssist performance), and absence of adverse effects such as pain or skin irritation. This comfort-based titration approach is consistent with published protocols for tSCS in SCI rehabilitation (Hofstoetter et al., 2015; Comino-Suárez et al., 2025; Suggitt et al., 2025). Session-level stimulation parameters are provided in Supplementary Table S1. Skin integrity and symptoms were monitored throughout each session.

## 5 Follow-up and outcomes

### 5.1 Outcome measures

Outcome assessments were performed at baseline, post-Phase I, post-Phase II, and at 4 weeks post-intervention. Neurological status was evaluated using ISNCSCI, including motor and sensory scoring. Functional mobility was assessed via WISCI-II, 10MWT for gait speed, and Spinal Cord Injury-Trunk Control Test (SCI-TCT). Spasticity was quantified using the MTS.

The primary outcome was gait speed as measured by the 10MWT, selected for its established responsiveness and MCID in SCI (Lam et al., 2008). Secondary outcomes included LEMS (ISNCSCI motor), spatiotemporal gait parameters (MobileGait), spasticity (MTS), electromyography (EMG) activation patterns, central motor conduction time (CMCT), and patient-reported outcomes (EQ-5D).

Surface EMG was recorded using the Cometa Wavetrack System (Cometa srl, Italy) with wireless Pico sensors during resting state and during sit-to-stand task. Bilateral recordings were obtained from the rectus abdominis, rectus femoris, biceps femoris, tibialis anterior, and lateral gastrocnemius. During resting assessments, the participant lay supine without movement for at least 10 s. Sit-to-stand trials were standardized with the participant seated on a chair with a fixed height of 45 cm, feet positioned flat on the floor, knees flexed at 90° and thighs parallel to the floor. Raw EMG data were processed by EMGandMotionTools software using a standard methodology. The data were high-pass filtered at 20 Hz with nine-order Butterworth filter, and low-pass filtered at 500 Hz with eight-order Butterworth filter. The EMG root-mean-square (RMS) values were calculated for each task condition (individual muscle activation and static standing) and subsequently normalized to the EMG RMS value obtained during the resting state.

CMCT was calculated to assess corticospinal tract integrity using transcranial magnetic stimulation (TMS) with motor evoked potentials (MEPs) recorded from bilateral abductor hallucis (Fujimoto et al., 2017; Funaba et al., 2021; Udupa and Chen, 2013). CMCT was assessed from the abductor hallucis (a lower limb muscle innervated by the S1–S2 segments, MRC 3/5), ensuring direct relevance to the lower extremity intervention. The abductor hallucis was selected as the target muscle for CMCT measurement as it is a standard recording site for lower limb MEPs in corticospinal tract assessment, with well-established normative data and methodological protocols (Fujimoto et al., 2017; Udupa and Chen, 2013). While the tibialis anterior and extensor hallucis longus are more directly involved in gait-related foot clearance.

#### 5.1.1 Data analysis

Given the single-case design, inferential statistics were not applied. Data are presented descriptively, with changes interpreted against established minimal clinically important differences (MCIDs) as benchmarks for clinical significance where available. Changes in outcomes are interpreted against established MCIDs where available: 0.13 m/s for gait speed on the 10MWT (Lam et al., 2008). No universally established MCID for LEMS in chronic incomplete SCI currently exists; therefore, motor score changes are interpreted descriptively in the context of individual myotome-level gains. Visual analysis of session-level data (gait speed, cadence, MinAssist) was used to evaluate within-phase trends.

### 5.2 Neurological outcomes

Progressive neurological improvement was observed throughout the intervention (Table 1). The neurological level of injury improved from T5 at baseline to T6 by post-Phase II, attributable to improved sensory recovery. The ASIA Impairment Scale grade remained D throughout the study, consistent with the classification at initial injury. Motor gains were predominantly right-sided, with the right lower extremity motor score improving from 19/25 at baseline to 23/25 post-Phase I (maintained post-Phase II), while the left improved from 16/25 to 18/25 post-Phase I and 19/25 post-Phase II. Motor gains were most pronounced in the right lower limb, particularly in ankle dorsiflexors (L4: 3 to 5) and long toe extensors (L5: 4 to 5), with gradual improvements in the left lower limb (L5: 0 to 2). Notably, right quadriceps (L3) improved from 4/5 to 5/5 by post-Phase I and was maintained. Left quadriceps remained at 5/5 throughout.

Light touch sensation improved symmetrically from 49/56 bilaterally at baseline to 51/56 bilaterally by post-Phase I, with total scores rising from 98/112 to 102/112 and remaining stable thereafter. Pinprick sensation showed continued improvement, particularly on the left side (43/56 to 48/56 across the study), with total scores increasing progressively from 86/112 at baseline to 91/112 post-Phase I and 95/112 post-Phase II, suggesting ongoing sensory recovery with the addition of tSCS.

Spasticity showed variable responses during the intervention (Table 1). Right hamstring spasticity (MTS R2–R1) remained at 50–53 from baseline through post-Phase II but resolved to 0 at follow-up. Left hamstring spasticity decreased from 20 at baseline to 15 post-Phase II and 0 at follow-up. Gastrocnemius spasticity showed initial increases (right: 32 to 40 post-Phase I; left: 25 to 30) before decreasing at follow-up (right: 17; left: 13). Left quadriceps spasticity resolved from 12 at baseline to 0 post-Phase I and remained at 0. The participant reported reducing his spasticity medication (Baclofen, from 65 mg to 55 mg daily) during Phase II of the study period. Given the concurrent medication reduction, the observed improvements in spasticity, particularly at the four-week follow-up, must be interpreted with caution, as disentangling the contribution of the intervention from the medication change is not possible in this single-case design. The medication reduction was patient-initiated based on subjective improvement and was not part of the study protocol. At the four-week follow-up, substantial improvements in spasticity were observed across all measured muscle groups.

### 5.3 Functional and gait outcomes

During Phase I, the participant completed an average of 765 steps per session (range: 403–905), covering a mean distance of 194.1 meters at an average training speed of 0.11 m/s. The variability in training speed observed toward the end of Phase I (sessions 13–16) was attributable to progressive reduction in robotic assistance, which required the participant to generate greater voluntary effort at the cost of walking speed. As MinAssist values decreased, the participant assumed a larger proportion of the gait effort, resulting in slower but more active stepping. Session-level data including steps taken, distance covered, speed, and duration are provided in Supplementary Table S1.

Substantial improvements in gait speed were observed, with 10MWT performance increasing from 0.26 m/s at baseline to 0.29 m/s at post-Phase I, 0.37 m/s at post-Phase II, and 0.41 m/s at four-week follow-up. The total improvement of 0.15 m/s exceeded the minimal clinically important difference (MCID) of 0.13 m/s for gait speed in SCI (Lam et al., 2008). Despite these gains, gait speed remained markedly reduced compared to normative values for age-matched adults (~1.10 to 1.20 m/s) reported in a South-East Asian population (Lau et al., 2020), representing approximately 35% of expected normal performance. Spatiotemporal gait analysis revealed marked gains following the combined intervention. Cadence improved from 31.53 to 39.74 steps/min (+26.1%) on the right and from 33.29 to 38.67 steps/min (+16.2%) on the left but remained significantly below normative ranges (~105 to 115 steps/min). Stride length increased from 0.91 m (right) and 0.95 m (left) at baseline to 1.17 m bilaterally at follow-up (+28.6% right; +23.2% left), approaching normative values (~1.18 to 1.25 m). Stride times decreased from 3.81 s (right) and 3.61 s (left) to 3.02 s (right, −20.7%) and 3.10 s (left, −14.1%), remained approximately threefold higher than normal (~1.05 to 1.10 s). Step length asymmetry improved from 0.41 m (right) and 0.50 m (left) at baseline to 0.62 m (right) and 0.56 m (left) at follow-up, reflecting improved inter-limb symmetry. Overall, cadence improved bilaterally, with gains observed in both the stronger right limb and the weaker left limb, although values remained below normative ranges (Table 1).

**Figure 2:**
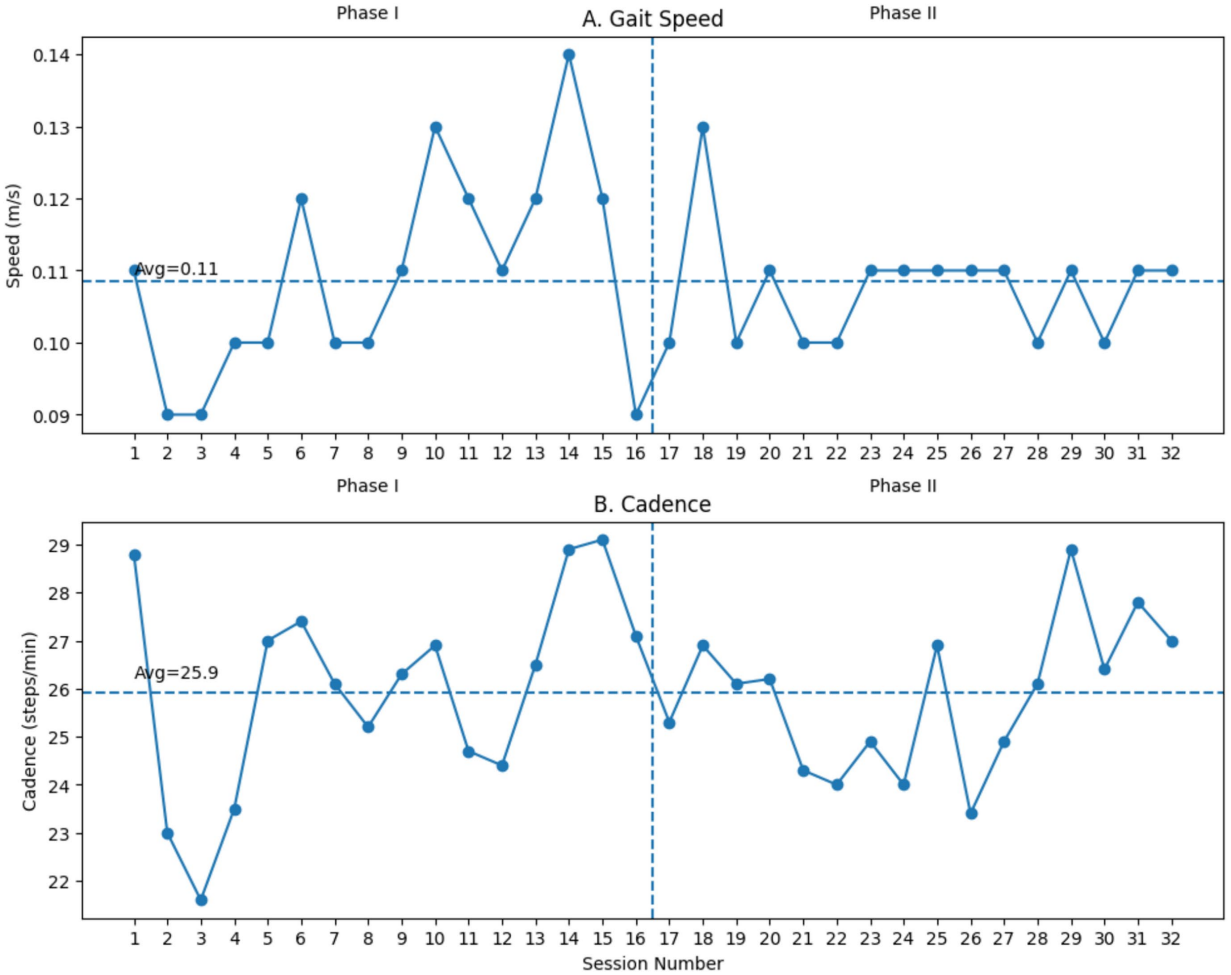
Sequential training outcomes across sessions: **(A)** Gait speed (m/s) and **(B)** cadence (steps/min) are displayed continuously from Session 1 to Session 32 to reflect the sequential study design. Phase I (Sessions 1–16) consisted of robotic-assisted gait training alone, while Phase II (Sessions 17–32) included concurrent transcutaneous spinal cord stimulation (tSCS). The dashed vertical line indicates the transition between phases. Horizontal dashed lines represent mean values across all sessions.

While average gait speed and cadence during training sessions were comparable between phases (0.11 m/s for both; cadence 26.0 vs. 25.7 steps/min) (Figure 2), there was a notable reduction in EksoGT assistance requirements in Phase II (average MinAssist: 50) compared to Phase I (average MinAssist: 61) (Figure 1), indicating greater active participant engagement with tSCS. tSCS stimulation amplitude was progressively titrated from 20 mA to 40 mA across Phase II sessions (Figure 1).

### 5.4 Neurophysiological outcomes

**Figure 3:**
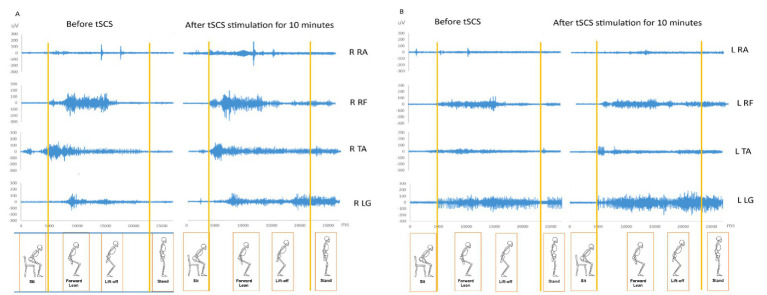
EMG activity in during sit-to-stand tasks before and after tSCS. The left panel **(A)** shows recordings from the right-sided muscles. The right panel **(B)** shows recordings from the right-sided muscles. R, Right; L, Left; RA, Rectus abdominis; RF, Rectus femoris; TA, Tibialis anterior; LG, Lateral gastrocnemius.

EMG recordings during sit-to-stand tasks were obtained at post-Phase II to evaluate the immediate effects of tSCS on neuromuscular activation. The participant performed two sit-to-stand trials before stimulation and two trials after 10 min of continuous tSCS at an intensity of 40 mA, during which the participant sat quietly. The participant required walking frame assistance during sit-to-stand transitions. Before stimulation, muscle activation patterns were inconsistent with weak bursts across proximal and distal muscles. Following tSCS, EMG activity became more organized with greater and more consistent muscle recruitment. Data from the best trials are presented in Figure 3 and Table 2. CMCT decreased from 21.9 ms to 19.7 ms on the right side following Phase II (Table 1).

**Table 2:** Overall EMG RMS during standing-up before and after tSCS stimulation for 10 min.

Overall EMG RMS	Before tSCS	After tSCS	Overall EMG RMS	Before tSCS	After tSCS
R Rectus Abdominis	3.68	6.97	L Rectus Abdominis	2.30	2.30
R Rectus Femoris	10.81	13.52	L Rectus Femoris	6.16	7.05
R Tibialis Anterior	8.12	8.91	L Tibialis Anterior	3.52	4.09
R Lateral Gastrocnemius	4.86	6.51	L Lateral Gastrocnemius	7.53	9.55

### 5.5 Quality of life and patient-reported outcomes

The EQ-5D-5L was selected as a measure of health-related quality of life as it has been validated and recommended for use in SCI populations (Jeawon et al., 2023). EQ-5D-5L dimension scores across all time points were as follows: Mobility improved from 4 (baseline) to 3 (post-Phase I, post-Phase II, and follow-up). Self-care improved from 2 (baseline) to 1 (post-Phase I and post-Phase II), returning to 2 at follow-up. Usual activities remained stable at 2 throughout. Pain/discomfort increased from 2 (baseline) to 3 (post-Phase I and post-Phase II) before returning to 2 at follow-up. Anxiety/depression remained at 1 (no problems) throughout. The EQ-VAS improved from 80 (baseline) to 90 (follow-up). The participant consistently reported low anxiety and depression (1) throughout the study and demonstrated improved health perception (visual analog scale: 80 to 90).

Post-training feedback was positive, with the participant citing subjective gains in strength, trunk control, walking ability, transfers, and reduced spasticity. The therapy schedule was considered appropriate, although the participant expressed a preference for more sessions per phase (up to 30, compared with the 16 delivered in each phase). The physiotherapist’s guidance was rated as excellent, no barriers to participation were reported, and the overall experience was rated as “very good.”

### 5.6 Tolerability and adverse events

The participant did not report any discomfort, pain, or intolerance related to tSCS at any stimulation intensity. No involuntary co-contractions, muscle spasms, or autonomic symptoms were observed during stimulation. The stimulation was well tolerated across all sessions, with no sessions discontinued or modified due to stimulation-related adverse effects. No adverse events attributable to tSCS therapy, overground RAGT training with EksoGT, or any study procedures were reported. Adverse event monitoring included assessment of skin integrity at electrode sites, pain (visual analog scale), blood pressure, heart rate, and screening for signs of autonomic dysreflexia before and after each session. The participant tolerated all interventions well, with no episodes of autonomic dysreflexia, skin breakdown, or other adverse effects throughout the study.

## 6 Discussion

This case report presents the first documented experience of integrating tSCS with overground exoskeletal training in SCI rehabilitation. The intervention was safe, well-tolerated, and associated with clinically meaningful improvements across multiple outcome domains.

### 6.1 Strengths of the approach

The substantial improvement in gait speed (0.26 to 0.41 m/s) exceeding the MCID of 0.13 m/s (Lam et al., 2008) suggests clinically meaningful functional gains. The improvement trajectory, with greater gains observed during Phase II (combined tSCS and RAGT) compared to Phase I (RAGT alone), supports the hypothesis of synergistic benefit. This aligns with emerging evidence that tSCS may enhance voluntary motor output when combined with task-specific training by increasing spinal excitability and facilitating activation of spared motor pathways (Gerasimenko et al., 2015; Hofstoetter et al., 2013, 2015). Gait speed exceeded the MCID of 0.13 m/s only at the four-week follow-up (0.41 m/s vs. baseline 0.26 m/s = +0.15 m/s), while at post-Phase II the improvement was 0.11 m/s. This delayed crossing of the MCID threshold may reflect continued consolidation of motor gains after cessation of training, a phenomenon observed in other neurorehabilitation studies (Gerasimenko et al., 2015; Moritz et al., 2024).

The motor gains observed in distal muscle groups (ankle dorsiflexors and toe extensors) are particularly noteworthy, as these functions are typically resistant to recovery in chronic SCI (Desrosiers et al., 2014). The concurrent improvements in sensation, particularly pinprick, suggest that the combined intervention may facilitate reactivation of spared afferent pathways and strengthen sensorimotor integration. Sensory feedback plays a critical role in refining gait mechanics and reducing reliance on compensatory strategies (Moreno-Lopez and Hollis, 2021).

The neurophysiological findings provide mechanistic support for the observed clinical improvements. The enhanced and more organized EMG activation patterns following tSCS, combined with the reduction in CMCT, are indicative of increased corticospinal excitability and improved motor pathway conduction. These findings are consistent with previous reports of neuromodulatory effects of non-invasive spinal stimulation techniques (Benavides et al., 2020; Knikou, 2014; Minassian et al., 2016). The proposed mechanism for the synergistic benefit of combined tSCS and RAGT involves the convergence of top-down (cortical) and bottom-up (spinal) inputs. tSCS is thought to modulate the excitability of spinal interneuronal networks, bringing subthreshold motor neurons closer to firing threshold, thereby facilitating voluntary motor commands descending from supraspinal centers (Gerasimenko et al., 2015). When combined with the intensive, repetitive, task-specific sensorimotor input provided by RAGT, this heightened spinal excitability may promote activity-dependent neuroplasticity at both spinal and supraspinal levels, strengthening residual corticospinal connections and facilitating reorganisation of locomotor circuits (Benavides et al., 2020; Knikou, 2014). The reduced EksoGT assistance requirements during Phase II further corroborate the enhanced voluntary motor engagement facilitated by tSCS.

Our findings are consistent with previous studies demonstrating the potential of tSCS to augment motor function in SCI (Alashram et al., 2023; Hofstoetter et al., 2015; Sayenko et al., 2019; Shankar et al., 2025a, 2025b). Recent work by Comino-Suárez et al. (2025) demonstrated that tSCS combined with robotic-assisted body weight-supported treadmill training enhanced motor scores and gait recovery in incomplete SCI through a randomized controlled trial. However, to our knowledge, this is the first report combining tSCS with overground exoskeletal training. While the RCT by Comino-Suárez et al. (2025) provides strong group-level evidence for tSCS combined with treadmill-based RAGT, overground exoskeletal systems such as the EksoGT offer several distinct advantages that warrant separate investigation. First, overground training requires active postural control and trunk engagement that treadmill-based body-weight-supported systems partially offload, potentially enhancing trunk stability and balance recovery (Alamro et al., 2018). Second, overground exoskeletons expose users to variable environmental challenges including turns, obstacles, and different floor surfaces, which may better facilitate transfer of motor gains to real-world community ambulation (Gil-Agudo et al., 2023). Third, full weight-bearing during overground walking generates physiological ground reaction forces that more closely replicate natural gait biomechanics, which may amplify the activity-dependent neuroplasticity promoted by concurrent tSCS (Gil-Agudo et al., 2023; Park et al., 2024). Fourth, the ecological validity of overground training may improve patient motivation and engagement, as evidenced by our participant’s high adherence (100%) and preference for additional sessions. The reduced MinAssist requirements observed in Phase II (average 50 vs. 61 in Phase I), despite comparable training speeds, suggest that tSCS may be particularly effective at facilitating voluntary motor engagement during the more demanding overground paradigm. These complementary advantages highlight the importance of evaluating tSCS across different RAGT platforms, as the optimal combination may depend on the specific rehabilitation goals and functional level of the individual patient. Nonetheless, we acknowledge that the present single-case design does not permit direct comparison with treadmill-based approaches, and adequately powered head-to-head trials comparing overground and treadmill-based RAGT combined with tSCS are warranted.

The improvement in trunk control observed during RAGT is consistent with studies demonstrating trunk muscle activation during exoskeleton use (Alamro et al., 2018) and the potential for tSCS to enhance trunk stability (Rath et al., 2018; Tharu et al., 2022). Although the SCI-TCT score remained at ceiling (24/24) throughout the study, suggesting no measurable change in trunk control on this instrument, the participant subjectively reported improved trunk stability. This discrepancy likely reflects a ceiling effect of the SCI-TCT in this relatively high-functioning participant (ASIA D). EMG recordings of the rectus abdominis during sit-to-stand tasks did show enhanced activation following tSCS, which may underlie the subjective improvement. We acknowledge that two sit-to-stand trials per condition is a limitation; future studies should include a minimum of three trials to improve reliability of pre-post comparisons. Although the standardised starting position was maintained, trunk flexion angle was not independently measured.

The spasticity findings warrant consideration. Right hamstring spasticity (MTS R2–R1) remained unchanged at 50 during both intervention phases but resolved to 0 at the four-week follow-up. This delayed improvement may reflect cumulative neuromodulatory and activity-dependent neuroplastic effects of transcutaneous spinal cord stimulation (tSCS). A systematic review by Anas R. Alashram and colleagues reported that tSCS may reduce spasticity by modulating spinal reflex excitability through activation of dorsal root afferents and enhancement of inhibitory spinal circuits following spinal cord injury. In contrast, left gastrocnemius spasticity increased modestly from baseline (32) to post-Phase I (40), which may reflect transient changes in reflex excitability during early neuromodulation-assisted gait training. However, interpretation should be cautious as the concurrent reduction in antispasticity medication may have contributed to this observation. These findings highlight the variable and time-dependent nature of spasticity responses to tSCS and underscore the need for further controlled studies examining spasticity modulation during combined neuromodulation and robotic gait rehabilitation.

Patient-reported outcomes support the acceptability of the combined intervention. The transient increase in pain during active training phases is consistent with the musculoskeletal demands of intensive gait training and resolved by follow-up. Low and stable anxiety and depression scores throughout indicate the intervention was psychologically well-tolerated. Improvements in EQ-5D-5L mobility and self-care domains, alongside subjective reports of enhanced strength, walking ability, and ease of transfers, suggest meaningful participation-level gains. The participant’s preference for additional sessions (up to 30 per phase) reflects high acceptability, an important consideration for future trial design.

### 6.2 Take-away lessons and clinical implications

This case suggests that integrating tSCS with overground RAGT is feasible and safe in clinical settings. The findings support the potential for tSCS to provide additional benefit when conventional rehabilitation approaches reach a plateau, particularly in chronic SCI where recovery options are limited. The titration of stimulation amplitude guided by motor assistance requirements (MinAssist scores) offers a practical approach to individualizing therapy. Future research should employ randomized controlled designs with larger samples to validate these preliminary findings, optimize stimulation parameters, determine ideal patient selection criteria, and assess long-term outcomes and cost-effectiveness.

From a patient perspective, the combined intervention was well-tolerated and the participant reported meaningful subjective improvements in daily function. From a clinical perspective, this approach appears feasible in a rehabilitation setting with appropriate equipment and trained staff, though the resource requirements of concurrent exoskeletal and neuromodulation therapy should be considered. From a research perspective, these preliminary findings support the design of adequately powered RCTs comparing combined tSCS+RAGT with RAGT alone, with pre-specified primary outcomes, standardised stimulation protocols, and longer follow-up periods.

### 6.3 Limitations

Several limitations must be acknowledged. First, as a single-case study with sequential intervention phases, we cannot definitively attribute improvements to tSCS versus continued RAGT training, natural recovery, or learning effects. The non-randomized, unblinded design limits causal inference. Second, the concurrent reduction in spasticity medication represents a significant confounder, particularly for interpreting spasticity outcomes and potentially motor and gait improvements. A detailed medication log has been provided (Supplementary Table S2), but the independent contribution of medication changes cannot be isolated. Third, the participant’s home and community activity levels were not systematically logged during the study period, and any changes in out-of-session physical activity may have contributed to the observed gains. Fourth, stimulation parameters were titrated based on comfort rather than optimised through systematic dose-finding, and the lack of a standardised optimisation protocol limits the replicability of the stimulation approach. Fifth, the relatively short follow-up period (four weeks post-intervention) limits assessment of long-term durability of gains.

## 7 Patient perspective

The participant reported a positive overall experience with the combined intervention. He noted subjective improvements in lower limb strength, trunk stability, walking ability, and ease of transfers. The reduction in spasticity was particularly appreciated. While the participant found the therapy schedule manageable, he expressed a preference for more intensive training with a higher number of sessions. The physiotherapy guidance throughout the study was highly valued, and no barriers to participation were identified. The participant rated his overall experience as “very good” and expressed interest in continued access to similar interventions.

## Data Availability

The original contributions presented in the study are included in the article/Supplementary material, further inquiries can be directed to the corresponding author.
