# Supplementary material for: Case Report: Efficacy of transcutaneous spinal cord stimulation combined with overground robotic-assisted gait training in chronic incomplete spinal cord injury
Source: Front Hum Neurosci. 2026 May 18;20:1795068. doi: 10.3389/fnhum.2026.1795068 (PMC13222957; doi:10.3389/fnhum.2026.1795068)
Supplement: Supplementary file 1 [file Supplementary_File_1.docx]

**Supplementary Table S1**. Session-level data included MinAssist scores, steps taken, distance covered, walk time, and walking speed during Phase I and Phase II EksoGT training, as well as tSCS current amplitude utilised in each Phase II session.

| Phase | Session | Min Assist (Left) | Min Assist (Right) | No. of Steps | Distance Walked (metres) | Walk time (mins) | Cadence (steps/min) | Speed (metres/min) | tSCS Current (mA) |
| --- | --- | --- | --- | --- | --- | --- | --- | --- | --- |
| 1 | 1 | 88 | 82 | 403 | 90 | 14 | 28.8 | 6.4 | - |
| 1 | 2 | 59 | 59 | 735 | 180 | 32 | 23.0 | 5.6 | - |
| 1 | 3 | 55 | 55 | 735 | 180 | 34 | 21.6 | 5.3 | - |
| 1 | 4 | 56 | 54 | 823 | 210 | 35 | 23.5 | 6.0 | - |
| 1 | 5 | 64 | 59 | 783 | 180 | 29 | 27.0 | 6.2 | - |
| 1 | 6 | 56 | 59 | 905 | 240 | 33 | 27.4 | 7.3 | - |
| 1 | 7 | 64 | 64 | 756 | 180 | 29 | 26.1 | 6.2 | - |
| 1 | 8 | 59 | 54 | 755 | 180 | 30 | 25.2 | 6.0 | - |
| 1 | 9 | 64 | 59 | 842 | 210 | 32 | 26.3 | 6.6 | - |
| 1 | 10 | 59 | 59 | 727 | 210 | 27 | 26.9 | 7.8 | - |
| 1 | 11 | 59 | 59 | 840 | 240 | 34 | 24.7 | 7.1 | - |
| 1 | 12 | 64 | 59 | 561 | 150 | 23 | 24.4 | 6.5 | - |
| 1 | 13 | 59 | 55 | 849 | 225 | 32 | 26.5 | 7.0 | - |
| 1 | 14 | 64 | 64 | 809 | 240 | 28 | 28.9 | 8.6 | - |
| 1 | 15 | 60 | 60 | 844 | 210 | 29 | 29.1 | 7.2 | - |
| 1 | 16 | 59 | 54 | 868 | 180 | 32 | 27.1 | 5.6 | - |
| 2 | 1 | - | - | - | - | - | - | - | 20 |
| 2 | 2 | 59 | 54 | 760 | 180 | 30 | 25.3 | 6.0 | 20 |
| 2 | 3 | 55 | 51 | 727 | 210 | 27 | 26.9 | 7.8 | 20 |
| 2 | 4 | 54 | 49 | 886 | 210 | 34 | 26.1 | 6.2 | 20 |
| 2 | 5 | 54 | 49 | 838 | 210 | 32 | 26.2 | 6.6 | 25 |
| 2 | 6 | 54 | 49 | 825 | 210 | 34 | 24.3 | 6.2 | 25 |
| 2 | 7 | 56 | 49 | 815 | 210 | 34 | 24.0 | 6.2 | 25 |
| 2 | 8 | 56 | 49 | 821 | 210 | 33 | 24.9 | 6.4 | 30 |
| 2 | 9 | 54 | 50 | 768 | 210 | 32 | 24.0 | 6.6 | 30 |
| 2 | 10 | 54 | 45 | 943 | 240 | 35 | 26.9 | 6.9 | 30 |
| 2 | 11 | 54 | 52 | 725 | 210 | 31 | 23.4 | 6.8 | 35 |
| 2 | 12 | 54 | 39 | 920 | 240 | 37 | 24.9 | 6.5 | 35 |
| 2 | 13 | 51 | 41 | 888 | 210 | 34 | 26.1 | 6.2 | 35 |
| 2 | 14 | 54 | 49 | 896 | 210 | 31 | 28.9 | 6.8 | 35 |
| 2 | 15 | 49 | 41 | 896 | 210 | 34 | 26.4 | 6.2 | 40 |
| 2 | 16 | 48 | 36 | 917 | 210 | 33 | 27.8 | 6.4 | 40 |

Supplementary Table S2. Medication log

| Phase | Baclofen dose  (6am/12pm/6pm/11pm) | Comments |
| --- | --- | --- |
| Before and throughout Phase 1 | 20mg /15mg /10mg /20mg | Strict 6-hourly timing. 20mg every morning, 15mg at noon, 10mg at 6pm, 20mg at 11pm |
| Phase 2 (initial sessions) | 20mg /15mg /10mg /20mg |  |
| Phase 2 (later sessions) | 20mg /10mg /10mg /15mg | Participant self-reduced the evening dose |

The participant's other medications — Colecalciferol, Magnesium, Potassium Citrate, Rosuvastatin, Lactulose, Senna, and Vitamin B Complex — remained unchanged throughout the study period.
